# Sentinel inflammatory demyelinating lesions preceding primary CNS lymphoma

**DOI:** 10.1590/0004-282X-anp-2020-0394

**Published:** 2021-06-14

**Authors:** Danielle Mesquita TORRES, Milena Sales PITOMBEIRA, Igor Bessa SANTIAGO, Gabriela Joca MARTINS, Kellen Paiva FERMON, Daniel Gurgel Fernandes TAVORA, Fernanda Martins Maia CARVALHO

**Affiliations:** 1 Hospital Geral de Fortaleza, Serviço de Neurologia, Fortaleza CE, Brazil. Hospital Geral de Fortaleza Hospital Geral de Fortaleza Serviço de Neurologia Fortaleza CE Brazil; 2 Universidade de Fortaleza, Programa de Pós Graduação em Ciências Médicas, Fortaleza CE, Brazil. Universidade de Fortaleza Universidade de Fortaleza Programa de Pós Graduação em Ciências Médicas Fortaleza CE Brazil

**Figure 1.**
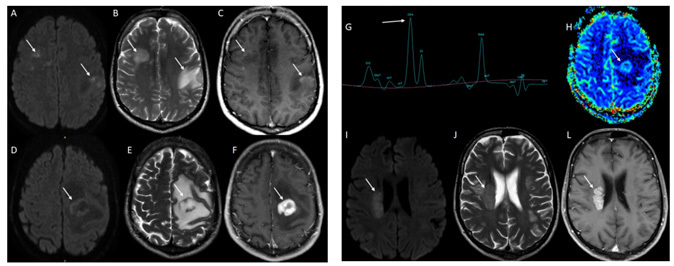
Magnetic resonance imaging exams. (A-C): first magnetic resonance imaging performed on March 2016 indicated diffusion restriction on diffusion-weighted image (A), T2 hypersignal (B), and peripheral enhancement on post-contrast T1 sequences (C). D-H: Neuroimaging performed on July 2018 showed a new lesion with peripheral restricted diffusion on diffusion-weighted image (D), T2 hypersignal (E), thick annular enhancement (F), spectroscopy revealed Cho peak increase (G) and minimal relative cerebral blood volume map (rCBV) increase. (H). (I-L): Brain magnetic resonance imaging performed on January 2019 disclosed a new right periventricular lesion with diffusion restriction on diffusion-weighted image (I), mild T2 hyperintensity sequences (J), and homogeneous contrast enhancement (L).

**Figure 2.**
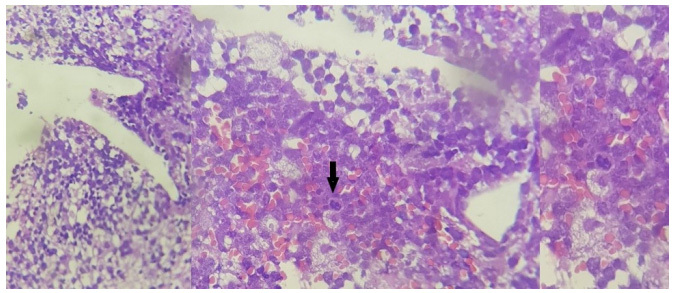
Histopathological examination showed atypical lymphoid cell proliferation.

A 29-year-old man presented with acute seizures and visual impairment. Brain magnetic resonance imaging (MRI) showed multiple white matter T2-lesions with incomplete peripheral enhancement (Figures 1A to 1C). Considering the hypothesis of acute disseminated encephalomyelitis, intravenous methylprednisolone (IVMP) was administrated with full recovery. Two years later, he presented right-sided weakness. MRI disclosed a new T2-lesion, and spectroscopy suggested a tumefactive inflammatory pattern (Figures 1D to 1H). New extensive cerebrospinal fluid (CSF) and blood workup, including aquaporin-4-IgG, was unremarkable. Partial improvement was observed following IVMP. Six months later, after new weakness in the left arm along with a new periventricular lesion (Figures 1I to 1L), a brain biopsy was performed. Histopathological analysis revealed primary central nervous system (CNS) lymphoma (Figure 2)^1^^,^^2^^,^^3^.
